# Impact of molecular quadrupole moments on the energy levels at organic heterojunctions

**DOI:** 10.1038/s41467-019-10435-2

**Published:** 2019-06-05

**Authors:** Martin Schwarze, Karl Sebastian Schellhammer, Katrin Ortstein, Johannes Benduhn, Christopher Gaul, Alexander Hinderhofer, Lorena Perdigón Toro, Reinhard Scholz, Jonas Kublitski, Steffen Roland, Matthias Lau, Carl Poelking, Denis Andrienko, Gianaurelio Cuniberti, Frank Schreiber, Dieter Neher, Koen Vandewal, Frank Ortmann, Karl Leo

**Affiliations:** 1 Dresden Integrated Center for Applied Physics and Photonic Materials (IAPP), Technische Universität Dresden, 01069 Dresden, Germany; 2 Institute for Materials Science, Max-Bergmann Center of Biomaterials and Dresden Center for Computational Materials Science, Technische Universität Dresden, 01069 Dresden, Germany; 3 Center for Advancing Electronics Dresden, Technische Universität Dresden, 01069 Dresden, Germany; 4 Institute of Applied Physics, University of Tübingen, Auf der Morgenstelle 10, 72076 Tübingen, Germany; 5 Institute of Physics and Astronomy, University of Potsdam, Karl-Liebknecht-Str. 24–25, 14476 Potsdam, Germany; 6 Max Planck Institute for Polymer Research, Ackermannweg 10, 55128 Mainz, Germany; 7 Present Address: Instituut voor Materiaalonderzoek (IMO), Hasselt University, Wetenschapspark 1, 3590 Diepenbeek, Belgium

**Keywords:** Electronic properties and materials, Semiconductors, Surfaces, interfaces and thin films, Solar cells, Organic molecules in materials science

## Abstract

The functionality of organic semiconductor devices crucially depends on molecular energies, namely the ionisation energy and the electron affinity. Ionisation energy and electron affinity values of thin films are, however, sensitive to film morphology and composition, making their prediction challenging. In a combined experimental and simulation study on zinc-phthalocyanine and its fluorinated derivatives, we show that changes in ionisation energy as a function of molecular orientation in neat films or mixing ratio in blends are proportional to the molecular quadrupole component along the π-π-stacking direction. We apply these findings to organic solar cells and demonstrate how the electrostatic interactions can be tuned to optimise the energy of the charge-transfer state at the donor−acceptor interface and the dissociation barrier for free charge carrier generation. The confirmation of the correlation between interfacial energies and quadrupole moments for other materials indicates its relevance for small molecules and polymers.

## Abstract (web summary)

The performance of organic semiconductor devices depends heavily on molecular parameters. Here, Schwarze et al. point out that the molecular quadrupole moment largely influences device energy levels and show how quadrupole moments can reduce the energy barrier for charge generation in solar cells.

## Introduction

Organic semiconductors gained much attention because of their attractive application in low-cost, large area, and flexible electronic devices^1–4^. While organic light-emitting diodes (OLEDs) already entered the market in thin film displays, several other promising applications such as solar cells, transistors, photodetectors, or lasers still require improvements in performance. In contrast to their inorganic counterparts, organic semiconductors typically consist of weakly bound molecules, where charge carriers occupy rather localised states. Associated to these states are the ionisation energy (IE) and electron affinity (EA) of organic molecules, which are related to the transport energies of holes and electrons and, consequently, determine the functionality of electronic devices^5,6^.

In organic solar cells (OSCs), fundamental processes determining the device performance are the dissociation of charge-transfer (CT) states at the donor−acceptor interface into free charges and their non-geminate recombination via CT states back to the ground state^7–9^. Therefore, the CT state energy (*E*_CT_) determines the open-circuit voltage (*V*_oc_) of OSCs^10^, while its difference to the energy of separated charges (*E*_CS_) influences the generation efficiency of free charge carriers and, thus, crucially affects both the short-circuit current density (*j*_sc_) and the fill-factor (FF)^11,12^. Both *E*_CT_ and *E*_CS_ are linked to IE of the donor and EA of the acceptor.

IE and EA of molecules in organic films significantly depend on molecular orientation and mixing ratio in blends^13–15^. In particular, charge−quadrupole interaction can induce large electrostatic shifts of the electronic levels in crystalline films^16–19^, which, for example, allows in blends with molecular intermixing, a continuous tuning of IE and EA by adjusting the ratio of two different molecular species^20,21^. Furthermore, simulations indicated that these interactions can assist the dissociation of CT states at planar donor−acceptor interfaces^22,23^. However, it remains an open question to which extent such findings for these model systems are general and, particularly, how they are applicable to donor:acceptor blends without long-range order which are usually employed in efficient OSCs.

In this study, we demonstrate the tunability of the solid-state IE by charge−quadrupole interactions and their relevance in systems with long-range and short-range order, being, thus, relevant for most organic devices. As a model system, we choose zinc-phthalocyanine (ZnPc) because of the possibility to gradually change its quadrupole moment (QPM) by stepwise fluorination (F_*n*_ZnPc)^20^. In order to establish the role of the quadrupole component perpendicular to the molecular plane (*Q*_π_), we measure for F_*n*_ZnPc the change in IE with molecular orientation, film thickness, and mixing ratio in blends. The ultraviolet photoelectron spectroscopy (UPS) analysis reveals a linear change of IE with *Q*_π_ in all cases. Moreover, when applying these findings to OSCs, we demonstrate how QPMs influence *E*_CT_ at planar and bulk heterojunctions between donor and acceptor. Time-delayed collection field (TDCF) measurements further show that electrostatic gradients induced by QPMs can assist free charge carrier generation in these solar cells. Finally, we extend the study to other material systems, indicating the relevance of the findings for a large variety of organic semiconductors.

## Results

### Dependence of thin flm energies on the component *Q*_π_

IE and EA of molecules in thin films deviate from their gas-phase values, IE_0_ and EA_0_, due to polarisation effects^24–26^. In weakly bound solids with localised states, the electrostatic corrections (Δ_+_ and Δ_−_) to the gas-phase values consist of an induced and a permanent contribution. While the first term decreases the distance between IE and EA, the latter originates from the interaction of excess charges with static charge distributions and shifts IE and EA equally^17–20^. We concentrate on the permanent contribution in this study, which is often dominated by the charge−quadrupole term in the multipole expansion because molecules with dipole moments often stack with alternating molecular orientations in ordered organic solids^16,18^. In this case, the permanent contribution can be approximated by a sum over the interaction energies of a charged molecule, described by its atomic excess charges *q*_*j*_ at positions **r**_*j*_, with the quadrupole tensors **Q**_*i*_ of all surrounding molecules at sites **r**_*i*_ ^27^:

1 $$E_{\mathrm{Q}} = {\mathop {\sum}\limits_{i,j}} \frac{q_{j}}{8 \pi \epsilon_{0}\epsilon_{\mathrm{r}}} \cdot \frac{\left({\mathbf{r}}_{i} - {\mathbf{r}}_{j} \right) \cdot {\mathbf{Q}}_{i} \cdot \left( {\mathbf{r}}_{i} - {\mathbf{r}}_{j} \right)}{\left| {\mathbf{r}}_{i} - {\mathbf{r}}_{j} \right|^5},$$

where we use the dielectric permittivity *ε*_r_ as a macroscopic constant. We describe the charged molecule by its actual distribution of atomic charges to have an appropriate description at distances in the range or below the spatial extent of molecules.

**Fig. 1:**
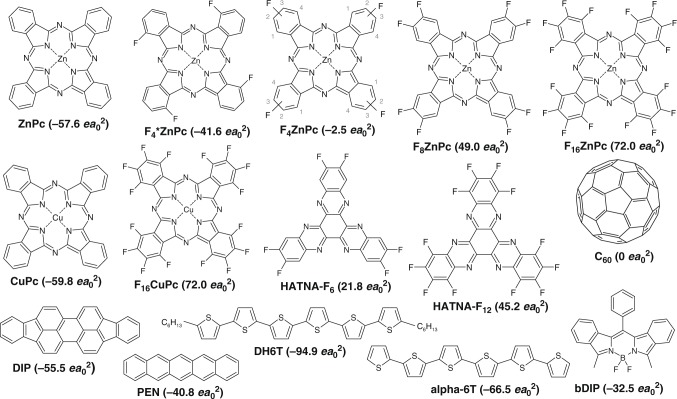
Chemical structures and quadrupole components of small molecules: Values in brackets are the quadrupole components perpendicular to the molecular plane (*Q*_π_), as calculated with density functional theory (DFT). Supplementary Table 1 summarises the in-plane quadrupole components. F_4_ZnPc is a mixture of different isomers, where the fluorine atom is bonded to one of the two outer carbon positions (2 or 3)^20^

**Fig. 2:**
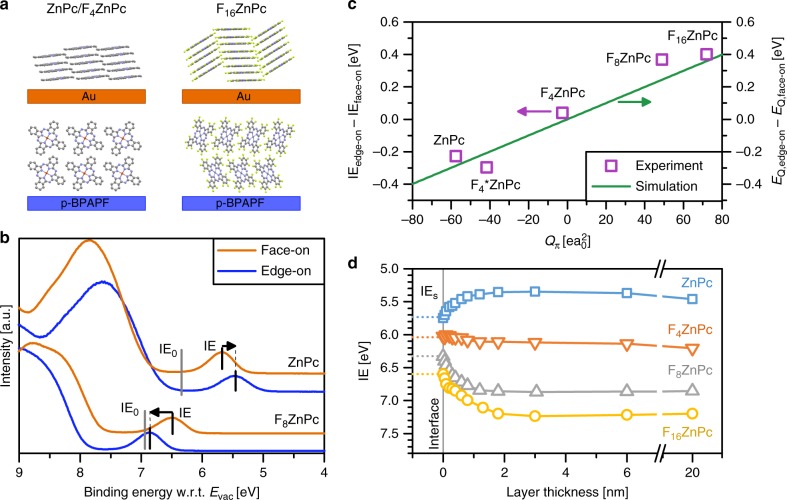
Dependence of IE on charge−quadrupole interactions along the π−π-stacking geometry: **a** Sketch of the film structure of ZnPc, F_4_ZnPc and F_16_ZnPc, showing the difference in molecular orientation between two different substrates. **b** UPS spectra of thin films of ZnPc (top) and F_8_ZnPc (bottom) in edge-on orientation (substrate: p-doped BPAPF on silver) and face-on orientation (substrate: gold). The different quadrupole moments of ZnPc and F_8_ZnPc cause electrostatic shifts in opposite directions from the gas-phase IE (IE_0_, obtained with DFT) to the IE value of thin films (obtained with UPS). **c** The difference in IE between edge-on and face-on orientation in UPS experiments (violet squares) scales with the calculated quadrupole component perpendicular to the molecular plane (*Q*_π_). The green solid line shows the difference in the charge−quadrupole interaction energy *E*_Q_ between both orientations, as obtained from simulation. **d** IE of F_*n*_ZnPc on p-doped BPAPF, obtained by subtracting the substrate spectrum from the superimposed spectra (see Supplementary Fig. 6a), changes strongly during the formation of the first monolayer in edge-on orientation due to charge−quadrupole interactions along the π−π-stacking direction. The slight changes of IE from 3 to 20 nm are also observed in simulation and can be explained by increased interactions with other quadrupole components than *Q*_π_. IE_s_ (dotted lines) is attributed to molecules with *E*_Q_ ≈ 0, being 0.6 eV smaller than IE_0_

Due to the strong dependence of *E*_Q_ on distance, *E*_Q_ changes with the chemical and crystal structure of the compound. Like many other planar molecules, F_*n*_ZnPc typically arranges in a π−π-stacking geometry^28,29^, where the intermolecular distance along the stacking direction (approximately 3.8 Å ^28^) is significantly smaller than along the other two directions (13−14.5 Å ^30^). Therefore, we first investigate if the quadrupole component perpendicular to the molecular plane (*Q*_π_) dominates *E*_Q_ (see Fig. 1 for *Q*_π_ values) by analysing for F_*n*_ZnPc layers (20 nm) the difference in IE between face-on and edge-on orientation of the molecules (Fig. 2a, see Supplementary Figs. 1–3 and Supplementary Note 1 for X-ray scattering results). The representative UPS spectra in Fig. 2b show that IE of a ZnPc film in edge-on orientation is 0.22 eV smaller as compared to the film in face-on orientation, in agreement with previous results^13^. In contrast, F_8_ZnPc exhibits an IE value in edge-on orientation that is 0.37 eV larger than the IE value in face-on orientation. To connect this behaviour to molecular properties, we calculate *Q*_π_ by density functional theory (DFT) for ZnPc and its fluorinated derivatives (results in Supplementary Table 1). As displayed in Fig. 2c, the difference in IE between edge-on and face-on orientation increases with *Q*_π_ from ZnPc to F_16_ZnPc.

To prove that *Q*_π_ dominates in *E*_Q_ (see Eq. 1), we calculate *E*_Q_ for a single charged molecule at the film surface for both orientations (see Methods and Supplementary Fig. 4). The difference in *E*_Q_ between both orientations exhibits the same correlation with *Q*_π_ as observed for the IE difference in experiment (Fig. 2c). Interestingly, the simulations reveal that the relevant range of charge−quadrupole interactions is different between both molecular orientations (Supplementary Fig. 5). For edge-on orientation, *E*_Q_ is dominated by interactions between the next few neighbours along the π−π-stacking geometry. For face-on orientation, *E*_Q_ is also dominated by interactions with *Q*_π_ components at small integration limits, which however is compensated by the interaction with other components when the integration limit in the lateral direction becomes larger than 100 nm. Previous investigations indicate that the interaction with other components than *Q*_π_ can be even more dominant in face-on orientation for molecules with a different symmetry than F_*n*_ZnPc such as pentacene, having two molecular short axes^19,31^. In the absence of long-range order, such as in donor:acceptor blends with crystal sizes much smaller than 100 nm, the interaction of charges with *Q*_π_ components should dominate^32–34^.

To further verify the dominance of charge−quadrupole interactions along the π−π-stacking direction for edge-on orientation, we measure IE of F_*n*_ZnPc for coverages below the monolayer thickness and evaporate the material stepwise onto p-doped BPAPF, leading to an edge-on orientation in thin films (Supplementary Figure 6). IE at very small coverages, denoted as IE_s_, deviates strongly from the value of thick layers (see dotted lines in Fig. 2d). Notably, for all ZnPc derivatives, IE_s_ is reduced by 0.6 eV compared to their respective gas-phase ionisation energies IE_0_, indicating minor influence of charge−quadrupole interactions on IE at low coverages. With increasing layer thickness, IE strongly changes and approaches the value of a thick edge-on oriented film when the monolayer thickness (13−14.5 Å ^30^) is reached. The strong IE shift can be explained by the formation of an ordered monolayer in edge-on orientation, leading to an increase of the magnitude of *E*_Q_ due to charge−quadrupole interactions along the π−π-stacking direction. In good agreement, the change of IE scales with the *Q*_π_ value of the respective ZnPc derivative (Supplementary Fig. 6c). Note that the change in IE until the first edge-on monolayer is formed can occur for different growth modes, such as island growth or transition of molecular orientation from face-on to edge-on.

After having demonstrated the strong effect of *Q*_π_ on the energy levels in model systems based on ZnPc derivatives, we further analyse its impact in donor:acceptor blends which are typically used in OSCs. Such blended structures usually exhibit significant structural disorder and phase separation that both can change with mixing ratio^13,32,35,36^. Previous studies found changes of energy levels with donor:acceptor mixing ratio, which were assigned to changes in crystal size and to electrostatic changes^13,15,37^. We trace back these energetic changes to the influence of the molecular tuning parameter *Q*_π_.

**Fig. 3:**
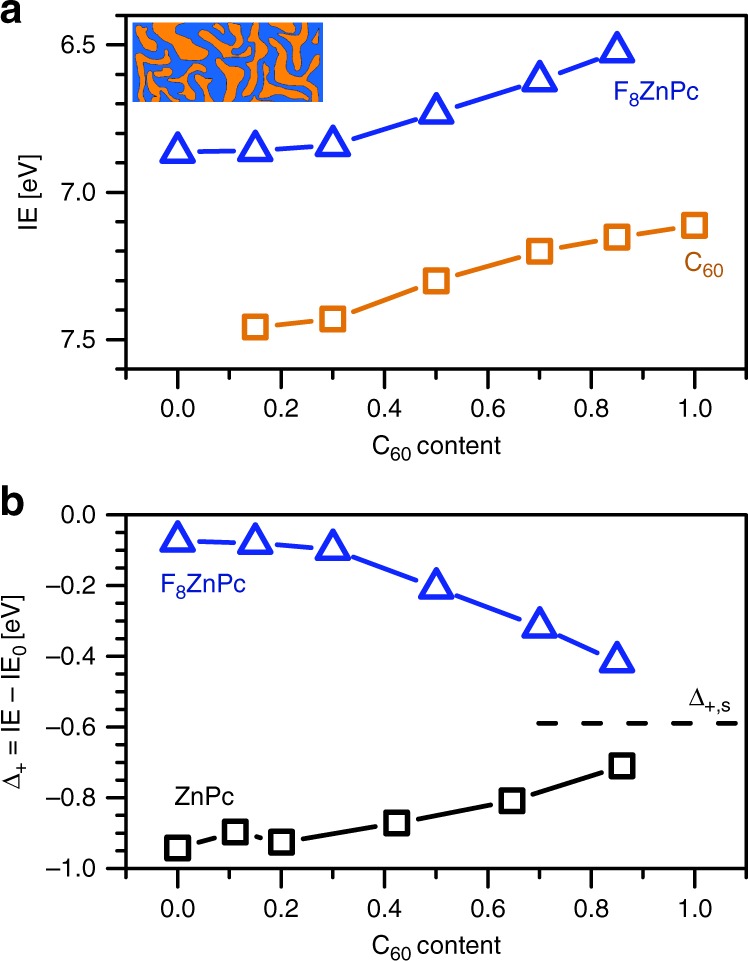
Electronic levels in donor:acceptor blends: **a** IE of F_8_ZnPc and C_60_ at different molar contents of C_60_, as obtained by UPS. **b** With increasing C_60_ content, the electrostatic corrections Δ_+_ of ZnPc and F_8_ZnPc, being the difference in IE between the thin film and the gas-phase, shift towards −0.6 eV. This value is expected for negligible charge−quadrupole interaction energy (see results in Fig. 2d for very small film coverages). Experimental IE values of ZnPc:C_60_ blends are taken from the study by Tietze et al.^13^

We measure the energy levels of F_8_ZnPc:C_60_ blends at different mixing ratios (see UPS spectra in Supplementary Fig. 7). As shown in Fig. 3a, IE of F_8_ZnPc decreases by more than 300 meV with increasing C_60_ content. IE of C_60_ shows a similar change, which suggests that a large amount of donor and acceptor molecules interact electrostatically with the other species despite the phase separation in these blends. The larger IE change of F_8_ZnPc at higher C_60_ contents indicates that F_8_ZnPc molecules which are closer to C_60_ molecules experience a larger electrostatic shift.

To validate that the electrostatic changes originate from charge−quadrupole interactions, we compare the behaviour of F_8_ZnPc:C_60_ blends to previously reported ZnPc:C_60_ blends^13^, as ZnPc and F_8_ZnPc exhibit a similar magnitude but a different sign of *Q*_π_. Figure 3b shows the electrostatic correction Δ_+_ of IE for ZnPc and F_8_ZnPc blended with C_60_. While Δ_+_ differs strongly between ZnPc and F_8_ZnPc in pure layers, this difference reduces continuously with increasing C_60_ concentration, and Δ_+_ finally approaches −0.6 eV. This value is also observed for very small coverages of (F_8_)ZnPc, indicating that the charge−quadrupole interaction energy *E*_Q_ approaches 0 for high C_60_ contents (see Fig. 2d for comparison). Therefore, we attribute the decrease (increase) of IE of F_8_ZnPc (ZnPc) with increasing C_60_ content to the interaction of charges with a reduced number of *Q*_π_ components of the donor.

The dominance of charge−quadrupole interactions along the π−π-stacking direction is attributed to the shorter intermolecular distance in this direction for the ZnPc derivatives. To verify whether this simple model is also valid for other materials, we extend our investigation to other molecules that show a strong dependence of IE on molecular orientation in neat films^13,14,38–41^ or on mixing ratio in donor:acceptor blends^15,37^ (see Fig. 1 for chemical structures). We calculate their QPMs and observe that for both cases the change in IE scales linearly with the respective *Q*_π_ (Supplementary Fig. 8). Notably, the slope is surprisingly similar to the one observed for intermixed blends of different ZnPc derivatives^20^ (Supplementary Fig. 9). Charge−quadrupole interactions are also relevant for polymers. Similar to sexithiophene (alpha-6T), poly-3-hexylthiophene (P3HT) shows a smaller IE for end-on orientation with the polymer chain perpendicular to the substrate plane^42^. This analysis indicates that the impact of *Q*_π_ on thin film energy levels is relevant for many organic materials including varying molecular structures and different morphologies of their (blend) films.

### Impact of *Q*_π_ at donor−acceptor interfaces in solar cells

**Fig. 4:**
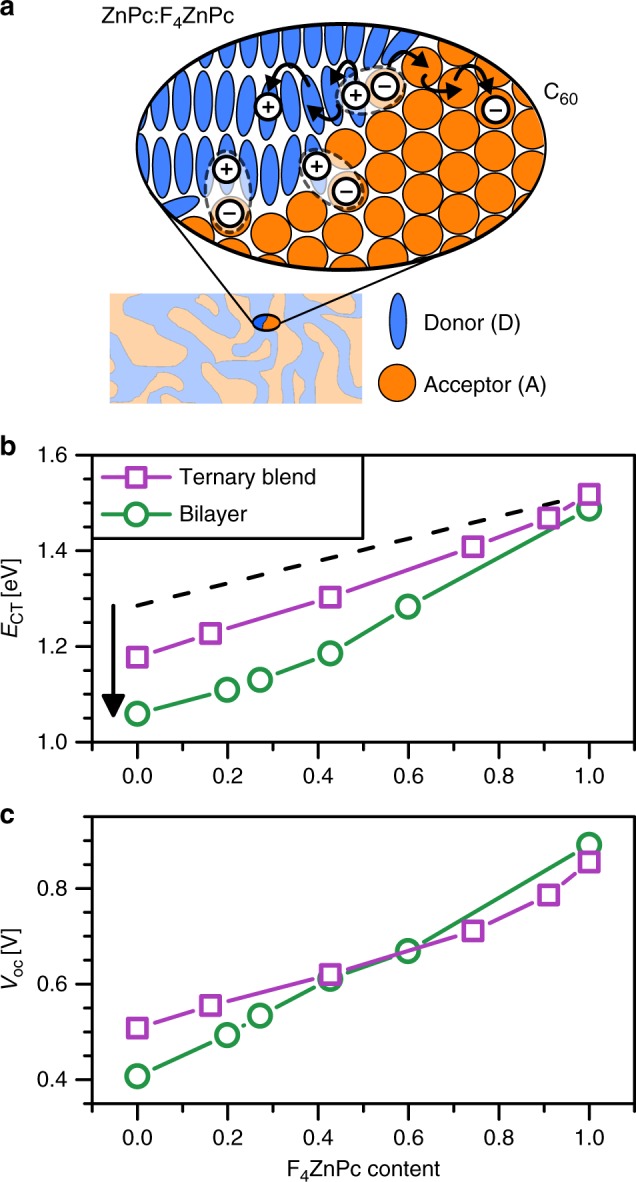
Charge-transfer state energies at bulk and planar heterojunctions: **a** Simplified sketch of the interface between donor and acceptor phase in a bulk heterojunction (BHJ). The black arrows sketch a representative dissociation process of a charge-transfer (CT) exciton. **b** Experimental change of *E*_CT_ as a function of the molar content of F_4_ZnPc in the intermixed donor phase (ZnPc:F_4_ZnPc), forming a ternary BHJ (purple squares) or a planar heterojunction (green circles) with the acceptor C_60_. *E*_CT_ values for the solar cells are obtained from Gaussian fits to sensitively measured external quantum efficiency (see Supplementary Fig. 11) and electroluminescence spectra^44^. The dashed black line is the shift of *E*_CT_, as calculated from the change of molecular parameters (see Eq. 4 in the Methods section). The black arrow highlights the decrease of *E*_CT_ at large ZnPc contents, which we attribute to charge−quadrupole interactions. **c** Change of *V*_oc_, measured at 1 sun illumination intensity, as a function of F_4_ZnPc content for both solar cell architectures. Note that the voltage losses (Δ*V*_oc_ = *E*_CT_/*e* − *V*_oc_) are slightly lower for planar heterojunctions due to the reduced interfacial area^58^

The functionality of OSCs is linked to the formation of charge-transfer (CT) states of a donor cation and an acceptor anion at their interface (Fig. 4a), mediating charge carrier dissociation and recombination in OSCs^7,8^. In the following, we demonstrate how molecular quadrupole moments can influence the CT state energy (*E*_CT_) as well as its difference to the energy of separated charges (*E*_CS_). For this purpose, we analyse ZnPc:F_4_ZnPc:C_60_ solar cells based on bulk heterojunctions (BHJ) with two electron donating molecules (ZnPc and F_4_ZnPc) and one acceptor (C_60_) with a fixed volume content of the acceptor (60%). Different mixing ratios between ZnPc and F_4_ZnPc are used to selectively change the average of the molecular parameters in the donor phase.

For analysing *E*_CT_, we obtain the relevant gas-phase energy levels of donor and acceptor molecules (IE_0,D_ and EA_0,A_) as well as the intramolecular relaxation energies of their ions by DFT. We further calculate for ZnPc/C_60_ and F_4_ZnPc/C_60_ the Coulomb binding energies of the energetically relaxed ion pairs and find that they differ up to several 10 meV between ZnPc and F_4_ZnPc because of their different charge distributions^43^ (see Methods section and Supplementary Fig. 10a). We calculate from these parameters the expected variation of *E*_CT_ from ZnPc:C_60_ to F_4_ZnPc:C_60_ (dashed line in Fig. 4b and Supplementary Fig. 10b). Experimental values of *E*_CT_ obtained from Gaussian fits to sensitively measured external quantum efficiency (see Supplementary Fig. 11) and electroluminescence spectra^44^. In good agreement with the calculations, the experimental values increase with F_4_ZnPc content (purple squares in Fig. 4b). However, the measured shift of *E*_CT_ is 0.1 eV larger than expected from the variation of molecular parameters, which can be partly attributed to the larger static energetic disorder at high ZnPc contents, reducing *E*_CT_ (Supplementary Fig. 12). In addition to energetic disorder, charge−quadrupole interactions lower the effective IE of the donor and further reduce *E*_CT_ at large amounts of ZnPc.

To verify the impact of charge−quadrupole interactions on *E*_CT_, we additionally fabricate solar cells with a planar heterojunction (PHJ) between a ZnPc:F_4_ZnPc blend layer with varying mixing ratio and a neat C_60_ layer. The ZnPc:F_4_ZnPc layer is grown on p-doped BPAPF to ensure edge-on orientation of the donor molecules, causing a large change of IE induced by charge−quadrupole interactions (see Fig. 2b and Supplementary Fig. 9). At high ZnPc contents, the planar devices exhibit a significantly lower *E*_CT_ (green circles in Fig. 4b) than the ternary BHJ devices. This can be explained by extended long-range order, which increases the charge−quadrupole interaction energy and reduces IE of the donor for large ZnPc contents. The change of *E*_CT_ with donor mixing ratio directly influences *V*_oc_ because charge carriers recombine via CT states at open-circuit^9^. Therefore, the different shifts of *E*_CT_ in BHJ and PHJ devices upon changing the donor content is reflected in a similar difference in *V*_oc_ shift, demonstrating the relevance of charge−quadrupole interactions for important device parameters (Fig. 4c).

**Fig. 5:**
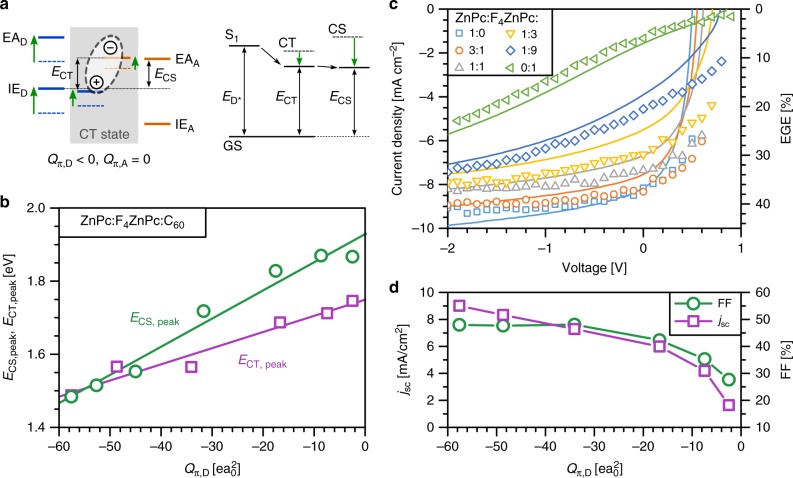
CT exciton dissociation facilitated by quadrupole moments: **a** Simplified sketch of the donor and acceptor energy levels at the interface (CT state), highlighted by the grey area, and in the bulk of donor and acceptor phase for *Q*_π,D_ = 0 (dashed lines) and *Q*_π,D_ < 0 (solid lines). The green arrows illustrate the energy shifts due to charge−quadrupole interactions, reducing the CT dissociation barrier (Δ*E*_diss_), i.e. the difference between the energy of separated charges (*E*_CS_) and *E*_CT_. The right panel sketches the change of E_CT_ and E_CS_ together with the singlet state (S_1_) energy and the ground state (GS). **b** *E*_CS,peak_, estimated from UPS measurements, increases stronger with the averaged *Q*_π,D_ as compared to the peak energy of the CT state (*E*_CT,peak_), indicating an enhancement of Δ*E*_diss_ with increasing *Q*_π,D_, i.e. with larger F_4_ZnPc contents. *E*_CS,peak_ and *E*_CT,peak_ are obtained from peak positions to exclude energetic disorder and experimental broadening from the analysis. Furthermore, *E*_CS,peak_ is reduced by 0.6 eV to account for the polarisation difference between surface and bulk^59,60^. The solid lines are guides to the eye. **c** Time-delayed collection field (TDCF) measurements show that the reduction in photocurrent and fill factor (FF) for large F_4_ZnPc contents is caused by field-dependent photocurrent generation. The solid lines and left axis show the current-density/voltage characteristics of the solar cells, whereas the symbols and right axis depict the external generation efficiency (EGE) of the photocurrent. **d** The increase of Δ*E*_diss_ with *Q*_π,D_, as shown in (**b**), causes a reduction of the short-circuit current density (*j*_sc_) and FF of the respective solar cells measured at 1 sun illumination conditions

The generation efficiency of photocurrent depends on the dissociation barrier of CT excitons (Δ*E*_diss_), which depends on the difference between *E*_CS_ and *E*_CT_^11^. We next discuss how quadrupole moments can induce electrostatic gradients at the donor−acceptor interface that lead to a lower Δ*E*_diss_. In the case of a negative quadrupole component *Q*_π,D_ of the donor, charge−quadrupole interactions along the π−π-stacking direction reduce IE_D_ and EA_D_ of donor molecules within the donor phase (green arrows in Fig. 5a). This shift is smaller at the interface with C_60_ due to the interaction with its neutral *Q*_π,A_ components. The negative *Q*_π,D_ influences the energies of acceptor molecules (IE_A_ and EA_A_) close to the interface, as indicated by the UPS measurements on donor−acceptor blends in Fig. 3. Therefore, the negative *Q*_π,D_ induces an electrostatic potential gradient from the donor to the acceptor phase, which directly reduces Δ*E*_diss_.

To analyse Δ*E*_diss_ in ZnPc:F_4_ZnPc:C_60_ solar cells, we obtain the difference between IE_D_ and EA_A_ in ternary blends with UPS and take this as an estimate for *E*_CS_^45^. The results in Fig. 5b show that *E*_CS_ increases more strongly with *Q*_π,D_ as compared to *E*_CT_, indicating a rise of Δ*E*_diss_ from high ZnPc to high F_4_ZnPc contents and verifying our considerations that charge−quadrupole interactions can reduce Δ*E*_diss_. For PHJs, we observe a similar increase of Δ*E*_diss_ for higher F_4_ZnPc contents (Supplementary Fig. 13). We perform TDCF measurements^11^ on ternary solar cells to investigate how the increase of Δ*E*_diss_ affects the generation efficiency of free charge carriers. In this method, donor molecules are excited by a short laser pulse. After a delay of 8 ns, when all geminate recombination has taken place^46,47^ (Supplementary Fig. 14), the charges are extracted by applying a large negative bias voltage. As shown in Fig. 5c, the amount of collected charges follows the current-density/voltage characteristics of the solar cells, revealing that the reduced photocurrent at high F_4_ZnPc contents is predominantly due to a field-dependent free charge carrier generation^11^. We attribute the increased field-dependence to the increased Δ*E*_diss_. The increase of Δ*E*_diss_ with *Q*_π,D_ causes a significant reduction of the device parameters FF and *j*_sc_ once *Q*_π,D_ is larger than −30 *ea*_0_^2^ (Fig. 5d). This finding explains the observation in previous studies where the use of F_4_*ZnPc (a variant of F_4_ZnPc, see Fig. 1 for the chemical structure) as donor in combination with C_60_ yields well-performing solar cells^48^. In contrast to F_4_ZnPc, F_4_*ZnPc has a more negative *Q*_π,D_ of −41.6 *ea*_0_^2^, being sufficiently low to ensure efficient CT exciton separation.

The correlation of FF and *j*_sc_ with *Q*_π_ is not restricted to this particular system. We further calculate *Q*_π,D_ and *Q*_π,A_ values for three donors (SubNc, ZnPc, F_4_*ZnPc) each combined with one non-fullerene acceptor (Cl_4_SubPc or Cl_6_SubPc). In PHJ devices^49^, FF and *j*_sc_ increase for all donors when the acceptor Cl_4_SubPc is substituted by Cl_6_SubPc, which can be explained by the increased difference between *Q*_π,D_ and *Q*_π,A_ (Supplementary Fig. 15). These results indicate that a precise adjustment of *Q*_π,A_ being larger than *Q*_π,D_ should be considered when designing efficient non-fullerene acceptor molecules. For example, *Q*_π,A_ can be increased by adding electron withdrawing side groups to the acceptor. In literature, there are already examples of efficient acceptors having such side groups^50,51^, where a larger degree of fluorination resulted in an improved charge extraction^52^. Furthermore, the results of a recent study indicate that quadrupole moments also affect polymer solar cells. Here, an improved charge generation in P3HT/PCBM bilayer solar cells was observed when P3HT was oriented end-on^42^, which can be explained by the reduced relevance of the positive quadrupole component along the polymer chain (see quadrupole components of alpha-6T in Supplementary Table 1 for comparison). Therefore, quadrupole moments and molecular orientation should be taken more into account for the design of future photovoltaic materials such as small molecules, oligomers and polymers.

## Discussion

In conclusion, we show that charge−quadrupole interactions along the π-π-stacking direction can induce large electrostatic energy shifts to the electronic levels of molecular films, depending on molecular orientation and blend composition. Due to the sensitivity of the interaction energy to the local morphology, the electronic levels of blends strongly depend on the mixing ratio. Utilising the example of OSCs, we show the dependency of the charge-transfer state energy at donor-acceptor interfaces on the quadrupole component in π−π-stacking direction. Moreover, we present a strategy to tune the driving force for free-charge-carrier generation by adjusting the respective quadrupole components, which can be used to optimise non-fullerene acceptor molecules. A similar correlation is found for other materials than ZnPc derivatives, suggesting that our findings can be applied to a large variety of small molecules and polymers. These results highlight the necessity to consider the quadrupole moment as an important molecular parameter in future material design for high-performing organic semiconductor devices.

## Methods

### Ultraviolet photoelectron spectroscopy

The spectra are acquired by a PHOIBOS 100 analyser system (Specs, Berlin, Germany) at a base pressure of 10^−11^ mbar using He I excitation lines (21.22 eV) and an energy resolution of around 150 meV. By repeating the sample production under the same experimental conditions, the experimental error for the position of the obtained energetic states is estimated to be 50 meV. The Fermi level positions of all spectra are calibrated to the Fermi edges of the gold or silver substrates. All samples are thermally (co-)evaporated at rates of 0.1–0.2 Å s^−1^ in UHV at a base pressure of 10^−8^ mbar using individual quartz crystal monitors for each material. As substrates, sputter-cleaned gold foils are used for face-on orientation and silver foils covered by 5 nm of an amorphous layer of p-doped BPAPF (3 wt%, doped with NDP9) for edge-on orientation^29^. NDP9 is a commercial p-dopant supplied by Novaled GmbH, Germany. The layer thickness of the organic layers under investigation is always 20 nm. IE values are obtained from the sum of the work function and the maximum position of the HOMO peak. The work function is extracted from the onset of the high binding energy cut-off.

### X-ray scattering

The experiments are performed at the ESRF, France (beamline ID03), with a photon energy of *E* = 22.0 keV. The reciprocal space maps (RSM) are measured under grazing incidence geometry with an angle of incidence of *α*_i_ = 0.07°. Each RSM is assembled from 16 single images recorded with a PILATUS 300k area detector. All measurements are performed in air. The samples are thermally (co-)evaporated at rates of 0.1−0.2 Å s^−1^ in UHV at a base pressure of 10^−8^ mbar using individual quartz crystal monitors for each material. As substrates, glass substrates covered by 1 nm of chromium and 30 nm of gold are used for face-on orientation and glass substrates covered by 5 nm of an amorphous BPAPF are used for edge-on orientation^29^. The layer thickness of the F_*n*_ZnPc layers is 20 nm.

### Solar cell device preparation

The solar cells are thermally evaporated at ultrahigh vacuum (base pressure < 10^−7^ mbar) on a glass substrate with a pre-structured indium tin oxide (ITO) contact (Thin Film Devices, USA). The layer stacks of the ternary bulk heterojunction solar cells are: Glass/ITO/BPAPF:NDP9 (40 nm, 5 wt%)/ZnPc:F_4_ZnPc (5 nm, varying ratio)/ZnPc:F_4_ZnPc:C_60_ (38 nm, varying ZnPc:F_4_ZnPc ratio, 60 vol% of C_60_)/C_60_ (15 nm)/C_60_:W_2_(hpp)_4_ (8 nm, 3 wt%)/Al (100 nm). The layer stacks for planar heterojunctions are: Glass/ ITO/ BPAPF:NDP9 (20 nm, 10 wt%)/ZnPc:F_4_ZnPc (10 nm, varying ratio)/C_60_ (40 nm)/BPhen (8 nm)/Al (100 nm). NDP9 is a commercial p-dopant supplied by Novaled GmbH, Germany. All the organic materials were purified 2−3 times by sublimation. The device area of 6.44 mm^2^ is defined by the geometrical overlap of the bottom and the top contact, verified with a profilometer. To avoid exposure to ambient conditions, the organic part of the device is covered by a small glass substrate which is glued on top. The relative content of donor and acceptor phases in ternary blends is estimated to have a precision of better than ±5 wt%.

### Current−voltage characteristics

The current−voltage characteristics in dark and under solar illumination are measured with a source measure unit (Keithley 2400, USA) at room temperature. For the latter condition, the solar cells are illuminated with a spectrally mismatch-corrected intensity of 100 mW cm^−2^ (AM1.5G) provided by a sun simulator (16 S-150 V.3 Solar Light Co., USA) and masked to avoid edge effects and to precisely define the area. The intensity is monitored with a calibrated Hamamatsu S1337 silicon photodiode.

### Sensitive external quantum efficiency (sEQE)

The light of a quartz halogen lamp (50 W) is chopped at 141 Hz and coupled into a monochromator (Newport Cornerstone 260 1/4m, USA). The resulting monochromatic light is focused onto the solar cell, of which the short-circuit current is fed to a current pre-amplifier before it is analysed with a lock-in amplifier (Signal Recovery 7280 DSP, USA). The time constant of the lock-in amplifier was chosen to be 0.5 s or 1.0 s and the amplification of the pre-amplifier was increased to resolve low photocurrents. The EQE is determined by dividing the photocurrent of the OSC by the flux of incoming photons, which was measured using a calibrated Si and InGaAs photodiode. The measurements are performed at room temperature.

### Electroluminescence (EL)

EL measurements were obtained at room temperature with an Andor SR393i-B spectrometer equipped with a cooled Si and cooled InGaAs CCD detector array (DU420A-BR-DD and DU491A-1.7, UK). The spectral response of the setup was calibrated with a reference lamp (Oriel 63355). The emission spectrum of the OSCs was recorded at different injection currents with respect to voltages, which were lower than or at least similar to the *V*_oc_ of the device at 1 sun illumination.

### Time-delayed collection field

In TDCF measurements, the device is excited while held at a varying pre-bias and after a delay time of 8 ns a collection bias of 2.5 V is applied. The excitation was generated with a diode-pumped, Q-switched Nd:YAG laser (NT242, EKSPLA, 500 Hz rep-rate, 5.2 ns pulse duration, 590 nm wavelength). Also, to compensate for the internal latency of the pulse generator, the laser pulse was delayed and homogeneously scattered in an 85 m long silica fibre (LEONI). An Agilent 81150A pulse generator was employed to apply the pre-bias and collection bias to a homebuilt amplifier directly connected to the sample. The current through the device was measured via a grounded 10 Ω resistor in series with the sample and recorded with an Agilent DSO9104H oscilloscope.

### Density functional theory

The simulations are performed to calculate the molecular quadrupole moments, the ionisation energy, and the intramolecular relaxation energy in gas-phase of the relevant molecules. The quadrupole tensor components *Q*_*ij*_ are obtained from following definition:

2 $$Q_{ij} = {\int} p ( {\mathbf{r}} ) \cdot \left( 3r_{i} r_{j} - |{\mathbf{r}}|^2 \delta_{ij} \right) \cdot {\mathbf{d}}^3 {\mathbf{r}}.$$

The ionisation energy was determined as the difference of the total energy of the positively charged molecule and the neutral molecule in the relaxed geometry of the neutral molecule. The intramolecular relaxation energy is determined as the difference in total energy between the negatively (positively) charged molecule in its optimised geometry and its energy in the geometry of the neutral molecule. We used the M06-2x exchange-correlation functional^53^ and the correlation-consistent basis set cc-pVTZ^54^ as implemented in the computational chemistry package NWChem^55^.

### Charge−quadrupole interaction energy calculation

The energy is calculated for a given molecule at site **r**_*j*_ as a discrete sum including all other molecules (at sites **r**_*i*_) in the considered geometry, according to

3 $$E_{\mathrm{Q}}\left( {\mathbf{r}}_{j} \right) = {\mathop{\sum }\limits_{i,k}} \frac{q_{j,k}}{8\pi \epsilon_{0} \epsilon_{\mathrm{r}}} \frac{\left( {\mathbf{r}}_{i} - {\mathbf{r}}_{j} - {\mathbf{\tau}}_{k} \right) \cdot {\mathbf{Q}}_{i} \cdot \left({\mathbf{r}}_{i} - {\mathbf{r}}_{j} - {\mathbf{\tau}}_{k} \right)}{\left| {\mathbf{r}}_{i} - {\mathbf{r}}_{j} - {\mathbf{\tau}}_{k} \right|^5}$$

with the quadrupole tensor **Q**_*i*_ of molecule *i* and the relative dielectric permittivity (assuming *ε*_r_ = 2.8^56^ for all F_*n*_ZnPcs). Hereby, *q*_*j,k*_ is the fractional excess charge at atom *k* of the molecule *j* and **r**_*j*_ + **τ**_*k*_ is its position. The quadrupole tensor and the fractional excess charges are obtained in gas phase for all molecules in their respective relaxed structures. The charge distributions and the resulting quadrupole moments might slightly differ in the film phase due to the surrounding polarisable medium. The film structure was generated according to the crystal structure of CuPc^57^ (see Supplementary Figs. 1–4). We assume a simplified orthorhombic lattice and take the intermolecular distances (approximately constant 3.8 Å and 13.5 Å) from literature^28,30^. For face-on geometry, a film thickness of 20 nm implies that we take into account 53 layers along the surface normal. We restrict the summation in lateral directions to a large area of 400 nm × 400 nm, which is sufficient for convergence. For edge-on geometry, we have 15 layers in the direction of the surface normal for a 20 nm film, while the lateral dimension of the integration region is equally big. To investigate the relevant range for the interaction energy, we vary the summation in lateral direction between 10 and 200 nm (Supplementary Fig. 5). In addition, we reduced the thickness of the film in edge-on orientation from 20 to 3 nm and observe an increase of the interaction energy by 10% for ZnPc, in good agreement to the change of IE observed in experiment (Fig. 2d).

### Energy change of CT states due to molecular parameters

We estimate the variation of the CT state energy (*E*_CT_) from the change of molecular parameters when replacing ZnPc with F_4_ZnPc using following expression:

4 $$E_{\mathrm{CT}} = {\mathrm{IE}}_{\mathrm{0,D}} - {\mathrm{EA}}_{\mathrm{0,A}} - E_{\mathrm{coul,CT}} - \lambda_{\mathrm{D}} - \lambda _{\mathrm{A}},$$

where IE_0,D_ is the gas-phase ionisation energy of the donor (ZnPc/F_4_ZnPc) and EA_0,A_ is the gas-phase electron affinity of the acceptor (C_60_). *E*_coul,CT_ is the Coulomb binding energy between the donor cation and the acceptor anion, which is screened by a mean dielectric constant of *ε*_r_ = 3.6, obtained from *ε*_r_ = 2.8 for ZnPc and *ε*_r_ = 2.8 for C_60_ ^56^. For a detailed description how *E*_coul,CT_ is obtained, we refer the reader to a our previous publication^43^. *λ*_D_ is the intramolecular relaxation energy of the donor cation and *λ*_A_ is the intramolecular relaxation energy of the acceptor anion. All values are obtained from DFT simulations. To compare with the experimental data in Fig. 4b, we additionally subtract a polarisation energy of 2.1 eV.

## Supplementary information

Supplementary Information

Peer Review File

## Data Availability

All the data supporting the findings of this study are available within the article, its Supplementary Information files, or from the corresponding authors upon reasonable request.

## Code availability

The code used for the calculation of the charge−quadrupole interaction energy is available from the corresponding authors upon reasonable request.
