# Supplementary material for: Impact of molecular quadrupole moments on the energy levels at organic heterojunctions
Source: Nat Commun. 2019 Jun 5;10:2466. doi: 10.1038/s41467-019-10435-2 (PMC6549189; doi:10.1038/s41467-019-10435-2)
Supplement: Supplementary file 1 — Supplementary Information [file 41467_2019_10435_MOESM1_ESM.pdf]

Supplementary Information for

"Impact of molecular quadrupole moments on the energy levels at organic heterojunctions"

By

Martin Schwarze, Karl Sebastian Schellhammer, Katrin Ortstein, Johannes Benduhn, Christopher Gaul, Alexander Hinderhofer, Lorena Perdigón Toro, Reinhard Scholz, Jonas Kublitski, Steffen Roland, Matthias Lau, Carl Poelking, Denis Andrienko, Gianaurelio Cuniberti, Frank Schreiber, Dieter Neher, Koen Vandewal, Frank Ortmann, Karl Leo

Supplementary Figures

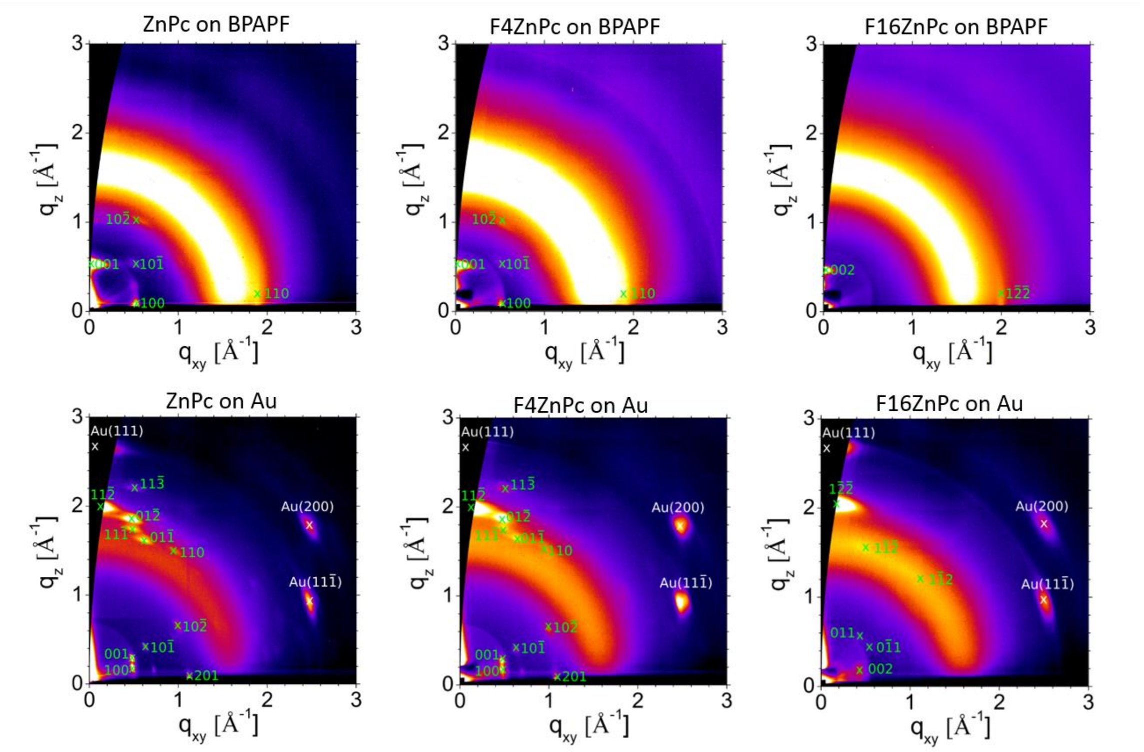

Supplementary Figure 1: Reciprocal space maps of ZnPc, F_4ZnPck, and F_{16}ZnPc on p-BPAPF (top row) and Au (bottom row).

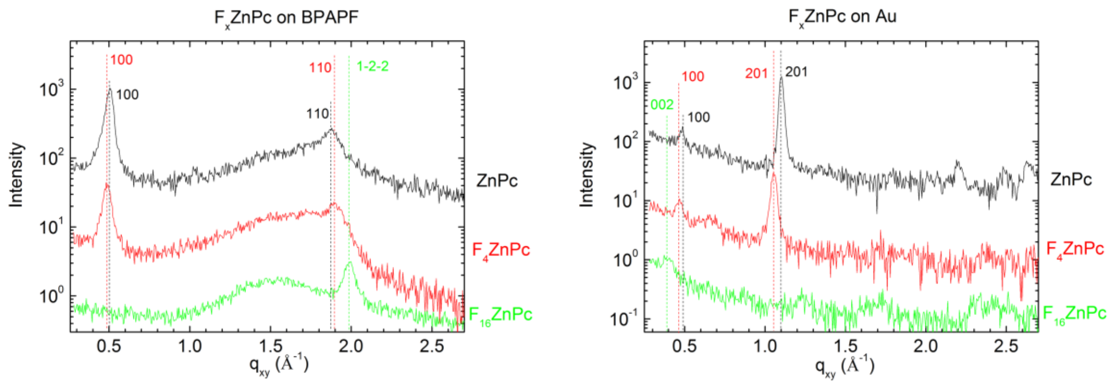

Supplementary Figure 2: GIXD of thin films on BPAPF (left) and Au (right).

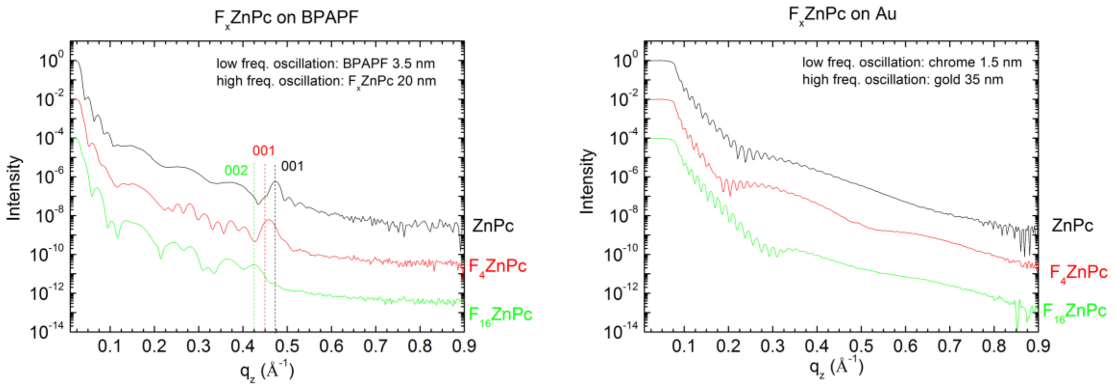

Supplementary Figure 3: X-ray reflectivity of thin films on BPAPF (left) and Au (right).

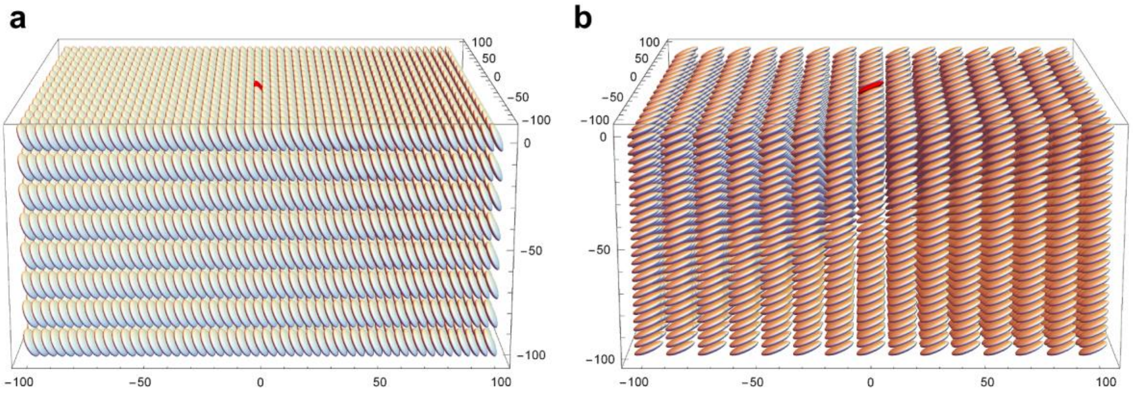

Supplementary Figure 4: Examples of film structures used for the calculations of the charge-quadrupole interaction energy (E_Q) of crystalline films in edge-on (a) and face-on orientation (b). The molecules are represented by discs for illustration purpose. The length scale is given in Å. The exact size of the film structures used in simulation is mentioned in the Method section. E_Q values are calculated for the red molecules at the film surface.

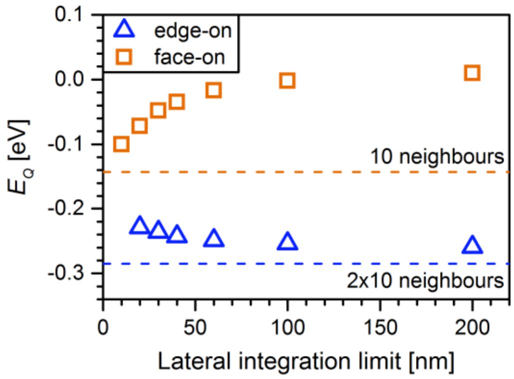

Supplementary Figure 5: Charge-quadrupole interaction energy from different integration domains: The dashed lines refer to a stack of ZnPc molecules, where the molecule under investigation has 10 neighbours on one side (orange) or 10 neighbours on each side (blue) in the π-π-stacking direction. The symbols (orange squares and blue triangles) are calculated for films with a thickness of 20 nm and different distances (20…200 nm) in the lateral direction parallel to the substrate plane. For edge-on orientation, the interaction energy of the films is dominated by the interaction along the π-π-stacking direction, *i.e.* the interaction with the quadrupole components Q_π. In contrast, for face-on orientation, the interaction with the other two quadrupole components becomes more important when the interaction range is large and compensates the interaction in π-π-stacking direction. In contrast to ZnPc, pentacene derivatives have two short axes and one long axis. Here, the permanent contribution to the electrostatic energy changes its sign for face-on orientation, which can be explained by the larger impact of the other components than Q_π, particular the other short axis component[3]. Pentacene quinones, where the largest quadrupole component is along the long axis, also show such a change of sign with molecular orientation[4]. This analysis indicates that the Q_π component dominates in edge-on orientation when it is the largest component and the π-π intermolecular distance is the shortest. In case of deviations from these properties, the effect becomes weaker or can even reverse, particular for face-on orientation or for molecules with more than one short axis.

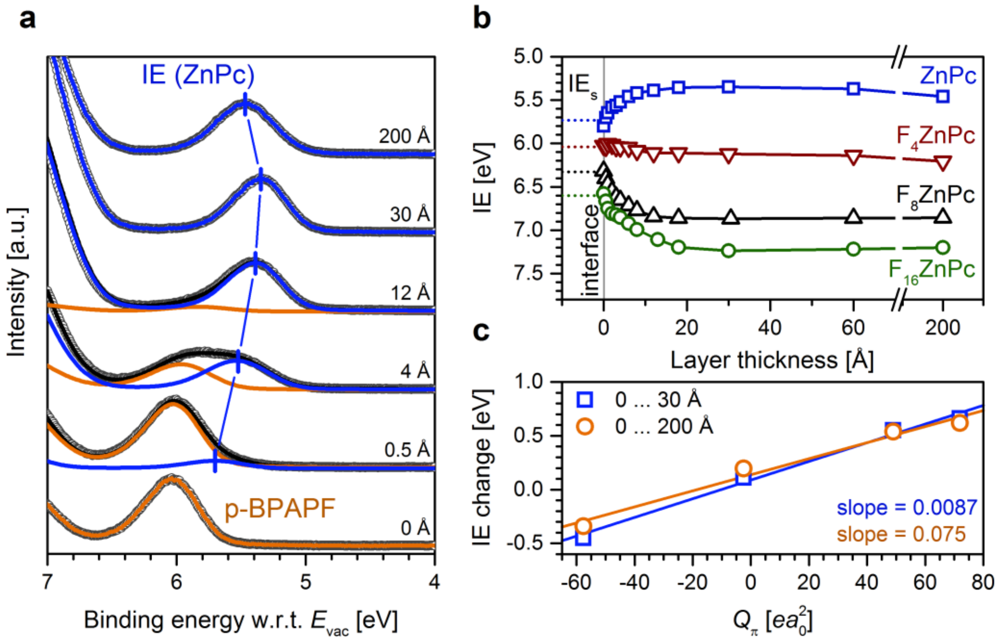

Supplementary Figure 6: Change of Electronic levels with film coverage: (a) UPS spectra of ZnPc in edge-on orientation at different layer thicknesses. Fitting of the spectra by a combination of the substrate spectrum and a thick ZnPc layer spectrum yields the respective ZnPc peak position for each thickness. (b) The formation of the first monolayer in edge-on orientation on the substrate yields a strong change of IE. IE$_S$ (indicated with dotted lines at the left side of the panel) are attributed to molecules with E_Q=0. (c) The change of IE within the first 30 Å and the first 200 Å scale with the respective quadrupole components Q_π of the F$_n$ZnPc component. The slightly lower slope for 200 Å can be explained by increased interactions with other quadrupole components than Q_π.

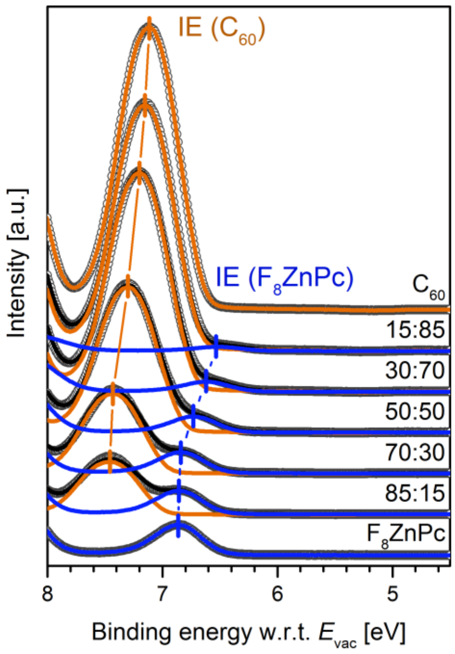

Supplementary Figure 7: Change of electronic levels in blends exhibiting phase separation: UPS spectra of F_8ZnPc:C_{60} blends at different mixing ratios, evaporated on p-doped BPAPF, are fitted by superimposing the spectra of neat F_8ZnPc and C_{60}. The F_8ZnPc and C_{60} peaks shift to lower binding energies with increasing C_{60} content.

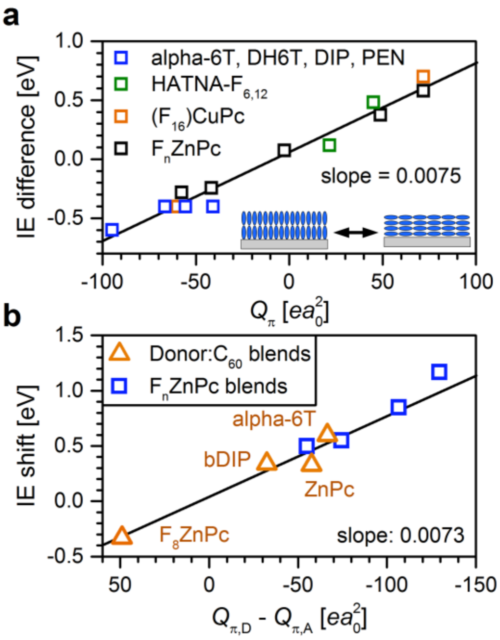

Supplementary Figure 8: Dependence of Q_π in other material systems: (a) The magnitude of Q_π, determined with DFT, determines the difference in IE between edge-on and face-on orientation. Note that the molecules alpha-6T, DH6T, DIP, and pentacene differ in their symmetry from F_nZnPc and have one long axis and two shorter axes. Even in such cases, the magnitude of Q_π seems to dominate the orientation dependence of IE in the crystalline films. While in edge-on orientation, surface molecules can interact along the two short axes with neighbouring molecules in all four directions within the surface plane, the interaction along the Q_π-axis is reduced for surface molecules in the case of face-on orientation, equally to the case of F_nZnPc. To compare with literature values, the onsets of the UPS HOMO peaks are taken to calculate IE. In contrast, IE values in the manuscript are obtained peak maxima. In Supplementary Table 1 and Supplementary Table 2, all values of the used materials are summarised[5,6,8,10,11,13–15]. (b) Difference in IE of four different donors between a neat crystalline film and a blend with a low donor content of around 10 vol% (orange triangles). Note that bDIP exhibits an additional dipole moment that, however, does not affect the general trend. The slope of the IE shifts in donor:C_{60} blends is only slightly lower than the slope observed in intermixed F_nZnPc blends[16] (blue squares, see also Supplementary Figure 9). Data is partly taken from literature[5,12] (Supplementary Table 2 and Supplementary Table 3).

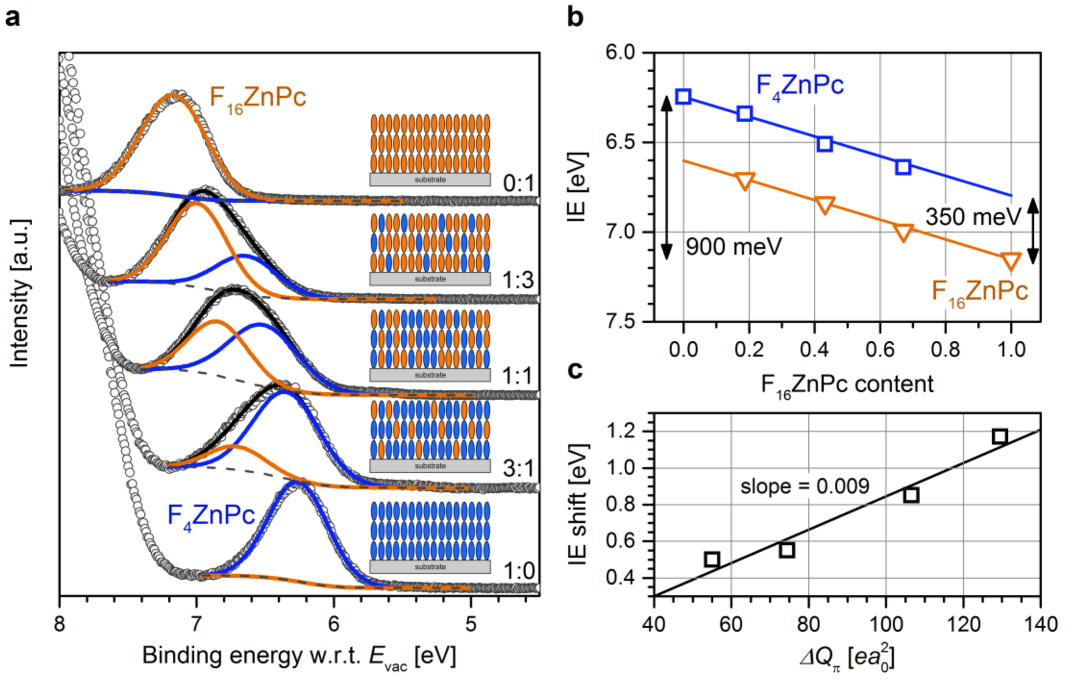

Supplementary Figure 9: Tuning of electronic levels by molecular intermixing: (a) UPS spectra of blends of F_4ZnPc and F_{16}ZnPc in edge-on orientation at different mixing ratios, which are illustrated by little sketches at the right side. The spectra are fitted by two Gaussian functions, one for each molecule[16]. (b) IE of F_4ZnPc and F_{16}ZnPc both shift linearly with the mixing ratio by 550 meV. (c) The complete IE change in intermixed blends scales with the difference between the respective values for the Q_π components. The experimental data for blends of ZnPc:F_4ZnPc, ZnPc:F_8ZnPc and ZnPc:F_{16}ZnPc are taken from previous work[16] (Supplementary Table 3).

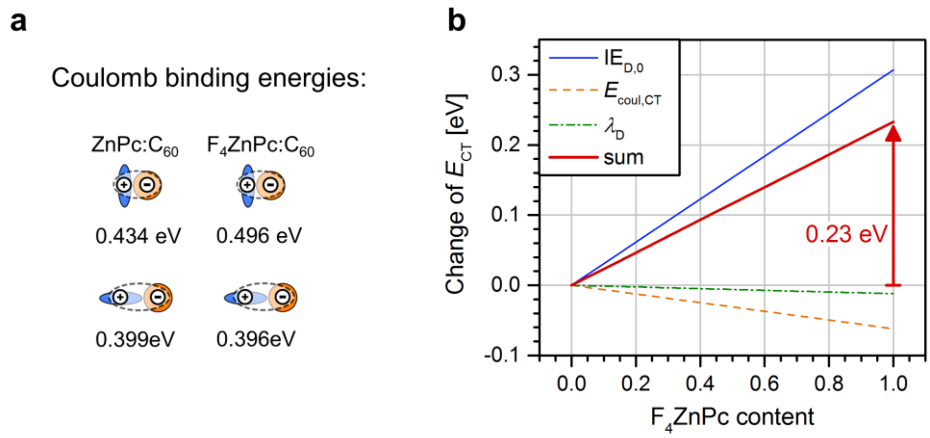

Supplementary Figure 10: Contributions to the change of E_{CT} for isolated CT states: (a) Simulated Coulomb binding energies of CT states with different relative molecular orientations of donor and acceptor molecule. The values are screened with a mean dielectric constant of $\varepsilon_r = 3.6$. The lower value for ZnPc:C_{60} in face-on orientation can be explained by the different charge distributions on ZnPc and F_4ZnPc[17]. (b) Expected change of E_{CT} for isolated CT states, when only the change in the Coulomb binding energy of the CT state ($E_{coul,CT}$) as well as the ionization energy ($IE_{0,D}$) and the relaxation energy (λ_D) of the donor molecule are considered. All values are calculated in gas phase by density functional theory (DFT).

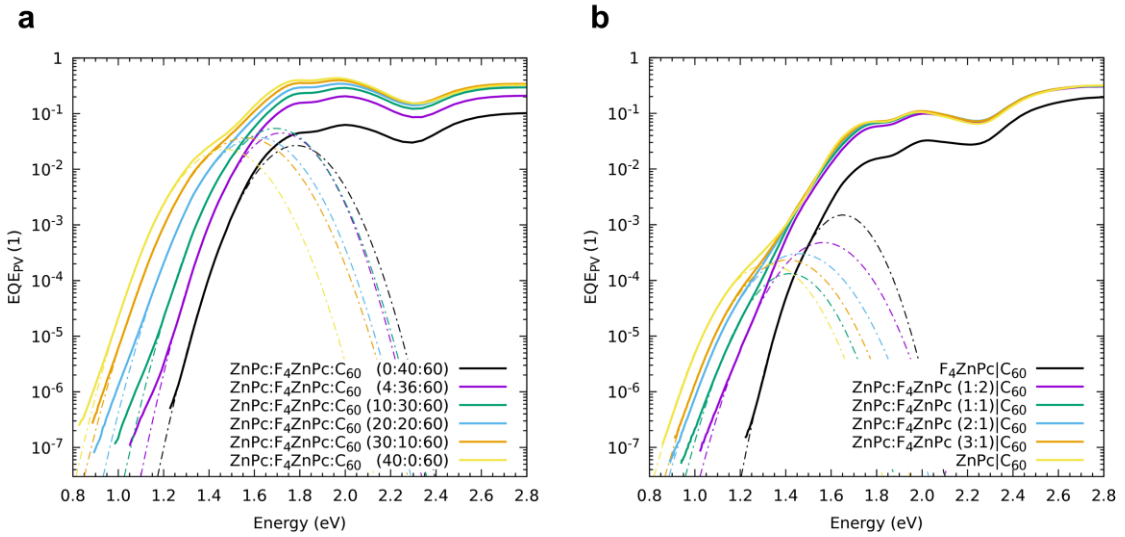

Supplementary Figure 11: Sensitive EQE spectra: Sensitive spectra of the external quantum efficiency of solar cells with a ternary bulk heterojunction (a) and a planar heterojunction (b) between donor (ZnPc:F_4ZnPc) and acceptor (C_{60}). The dashed lines are Gaussian fits to the spectra.

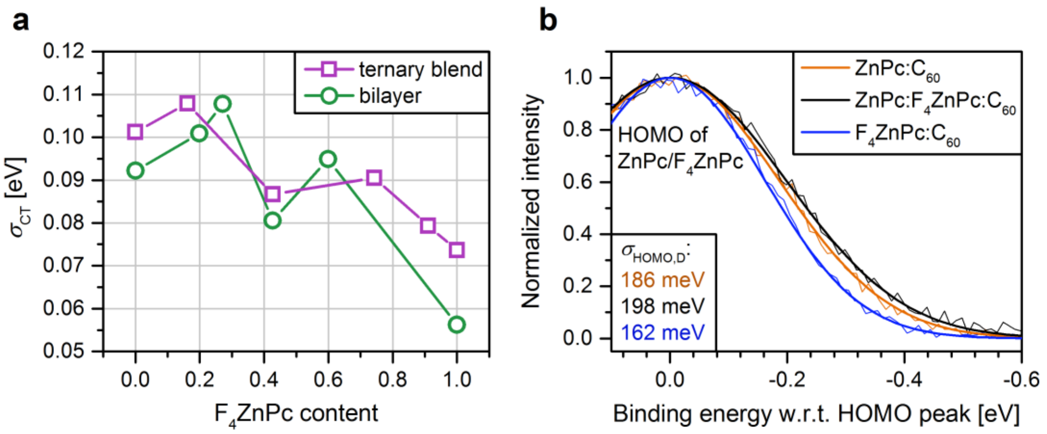

Supplementary Figure 12: Static energetic disorder of CT states: (a) The static energetic disorder (σ_{CT}) is obtained by subtracting the intramolecular relaxation energies of donor cation (λ_D) and acceptor anion (λ_A), obtained from DFT simulations, from the experimental peak width obtained from sensitive EQE spectra (σ_{exp}), based on following formula: $2k_BT\sigma_{exp} = \sigma_{CT}^2 + 2k_BT \cdot (\lambda_D + \lambda_A)$. (b) The UPS spectra show the HOMO peaks of ZnPc/F_4ZnPc of blends with C_{60}. The standard deviations of the donor HOMO peaks ($\sigma_{HOMO,D}$) are obtained from Gaussian fits to the spectra and are listed in the bottom left corner of the graph. Due to the large quadrupole moment of ZnPc, structural disorder such as variation in molecular orientations reflect in a local variation of the charge-quadrupole interaction energy and a larger energetic disorder in comparison to F_4ZnPc. This is not the case for F_4ZnPc because it exhibits Q_π close to zero.

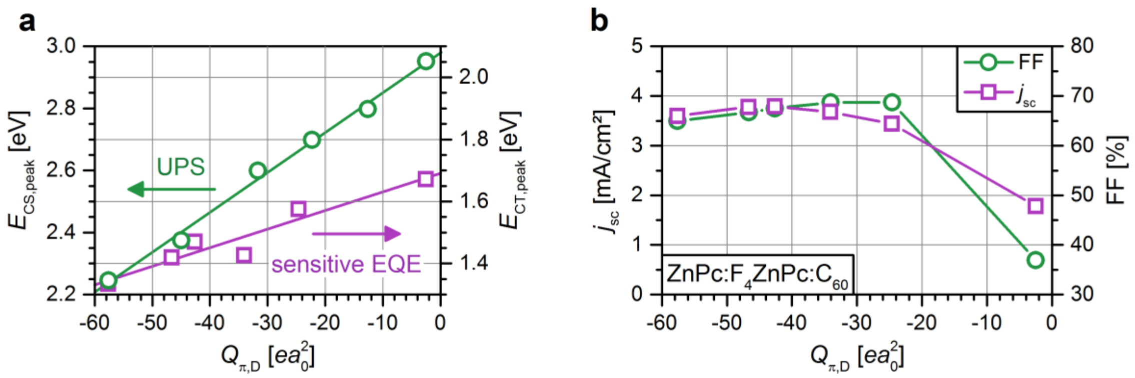

Supplementary Figure 13: Change of the CT exciton dissociation barrier at planar heterojunctions: (a) The difference between the energy of separated charges (E_{CS}) and the energy of the CT state (E_{CT}) increases with the mean out-of-plane quadrupole component of the donor ($Q_{\pi,D}$) by 0.4 eV. The peak positions are compared to exclude the impact of static energetic disorder on the CT dissociation barrier. (b) The increased dissociation barrier at high F_4ZnPc contents causes a decrease of fill factor (FF) and short-circuit current density (j_{sc}).

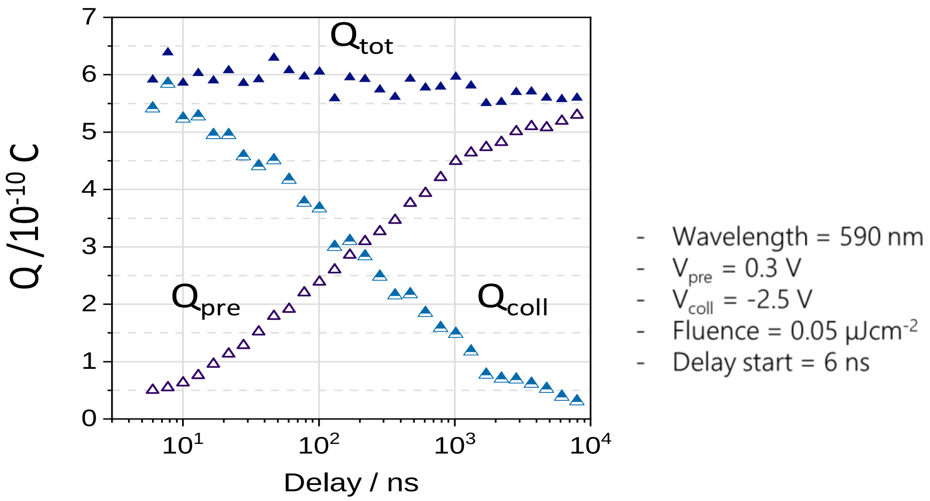

Supplementary Figure 14: TDCF experiment with varying delay times between excitation and extraction: Q_{pre} is the charge flowing out of the device during delay and Q_{coll} is the charge remaining in the device after the delay. Q_{tot} is the sum of both. In the case of significant geminate recombination of long-lived CT states, Q_{tot} would show an initial drop (because at longer times, there are less charges which can be extracted), which is apparently not the case.

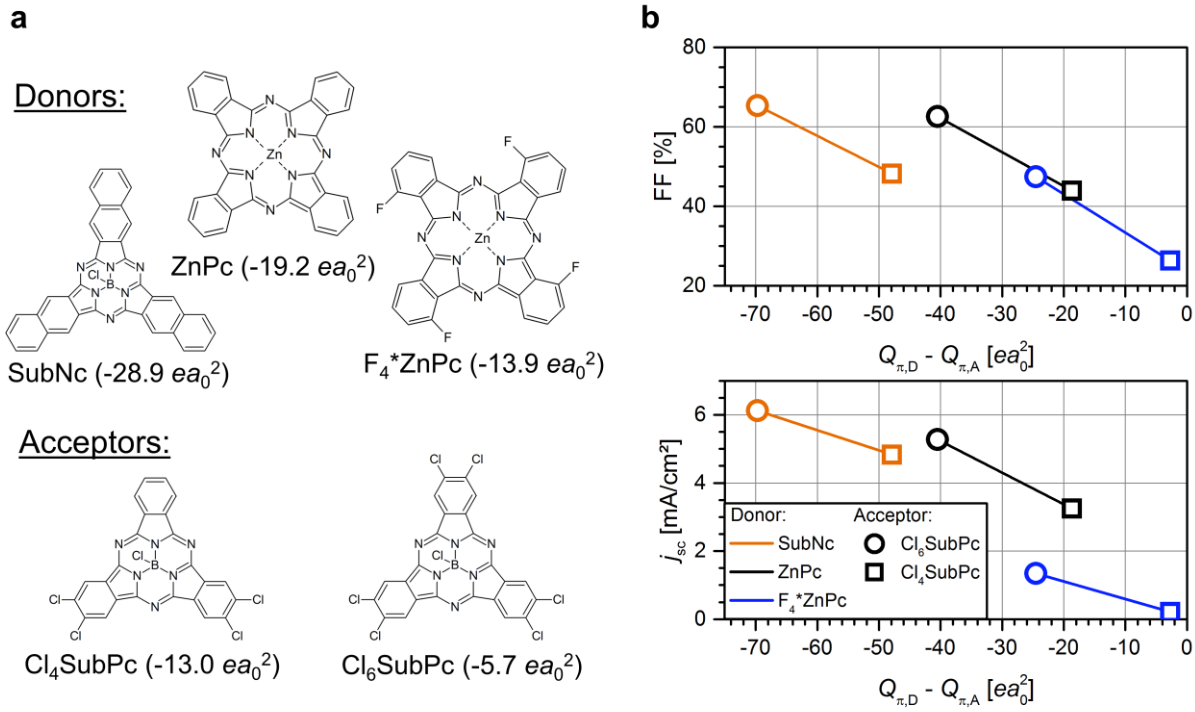

Supplementary Figure 15: Dependence of fill factor and short-circuit current density on quadrupole moments for other material systems: (a) Chemical structures of donor and non-fullerene acceptor molecules as well as their respective calculated quadrupole components Q_π. (b) The fill factor FF and the short-circuit current density j_{sc} changes with the difference of the respective Q_π values. The slight deviation of SubNc from ZnPc/F$_4$*ZnPc might be due to its large out-of-plane dipole moment. Note that the absorption of the different acceptor molecules is similar and cannot cause the change in j_{sc}[18]. The experimental data is taken from reference [18].

Supplementary Tables

Molecule	Q_1 (in plane) [ea_0^2]	Q_2 (in plane) [ea_0^2]	Q_π (out of plane) [ea_0^2]	IE_0 (gas phase) [eV]
ZnPc	28.8	28.8	-57.6	6.34
F_4*ZnPc	20.8	20.8	-41.6	6.54
F_4ZnPc	1.2	1.2	-2.5	6.65
F_8ZnPc	-24.5	-24.5	49.0	6.94
F_{16}ZnPc	-36.0	-36.0	72.0	7.21
C_{60}	0	0	0	8.09
HATNA-F_6	-10.9	-10.9	21.8	8.82
HATNA-F_{12}	-22.6	-22.6	45.2	9.25
CuPc	29.9	29.9	-59.8	8.78
F_{16}CuPc	-36.0	-36.0	72.0	9.77
PEN	18.0	22.8	-40.8	6.51
DIP	21.9	33.6	-55.5	7.01
alpha-6T	64.0	2.5	-66.5	6.70
DH6T	131.9	-37.0	-94.9	6.54
bDIP	23.9	8.6	-32.5	6.55
SubNc	43.7	43.1	-86.8	6.23
Cl_4SubPc	-17.0	55.9	-38.9	7.20
Cl_6SubPc	8.7	8.4	-17.1	7.28

Supplementary Table 1: Calculated quadrupole moments and ionisation energies: Anisotropic values of the molecular quadrupole moments are calculated by DFT, where Q_π is the component perpendicular to the molecular plane. Values for F_4ZnPc are mean values of all possible isomers. IE_0 is the calculated ionisation energy of a single molecule in gas-phase.

Molecule	IE onset (face-on) [eV]	IE onset (edge-on) [eV]	IE change (blend with C_{60}) [eV]
ZnPc	5.30	5.02	0.33 [5]
F_4*ZnPc	5.46 [6]	5.22	
F_4ZnPc	5.75	5.82	
F_8ZnPc	6.10	6.48	-0.31
F_{16}ZnPc	6.20 [7]	6.76	
HATNA-F_6	7.72 [8]	7.84	
HATNA-F_{12}	7.73 [8]	8.21	
CuPc	5.20 [9]	4.80 [9]	
F_{16}CuPc	5.90 [9]	6.60 [9]	
Pentacene	5.41	5.01	
DIP	5.60 [10]	5.20 [10]	
alpha-6T	5.40 [11]	5.00 [11]	0.60 [12]
DH6T	5.30 [11]	4.70 [11]	
bDIP			0.34 [12]

Supplementary Table 2: Summary of UPS energies: UPS measurements on thin films on strongly and weakly interacting substrates yield the ionisation energies (IE) in face-on and edge-on orientation. For a better comparison with literature values, the table shows onset values of the IE. The difference between IE of a pure donor film in edge-on orientation and IE of the donor within a blend with C_{60} (low donor content of around 10 vol%) yield the IE change in the last column. The IE change was obtained from the UPS onsets.

Molecule	IE difference (neat films) [eV]	IE difference (blends) [eV]	Q_π difference [ea_0^2]
$ZnPc{:}F_4ZnPc$	0.70 [16]	0.20 [16]	55.1
$ZnPc{:}F_8ZnPc$	1.19 [16]	0.34 [16]	106.6
$ZnPc{:}F_{16}ZnPc$	1.60 [16]	0.43 [16]	129.6
$F_4ZnPc{:}F_{16}ZnPc$	0.90	0.35	74.5

Supplementary Table 3: Tuning of electronic levels by molecular intermixing: IE difference between neat films in edge-on orientation (second column) and IE difference of the two materials in blends (third column). The last column shows the difference of the respective out-of-plane quadrupole components. Some of the experimental values are taken from literature.

Supplementary Notes

Supplementary Note 1:

Molecular structures on two different substrates (Au and p-BPAPF) are verified by X-ray scattering experiments. From the reciprocal space maps in Supplementary Figure 1, we determine that ZnPc and F_4ZnPc nucleate on both substrates in unit cells, very similar to the CuPc α-polymorph with one molecule per unit cell[1]. Bragg reflections from F_{16}ZnPc are consistent with the F_{16}ZnPc single crystal unit cell with two molecules per unit cell forming a herringbone structure[2]. We note that the intense broad rings in the reciprocal space maps are due to scattering from glass substrates and not from the organic thin films.

The molecular orientation of ZnPc, F_4ZnPc, and F_{16}ZnPc on p-BPAPF is nearly upright standing with the (001)/(002) plane parallel to substrate. In contrast, all three materials adopt a nearly flat lying orientation on Au substrates. While the (11-2) plane is parallel to the substrate for ZnPc/F_4ZnPc, the (1-2-2) plane is nearly parallel to the substrate for F_{16}ZnPc. Fig. 2a in the main text sketches the molecular orientations for both types of substrates.

Supplementary References

1. Hoshino, A., Takenaka, Y. & Miyaji, H. Redetermination of the crystal structure of α-copper phthalocyanine grown on KCl. *Acta Crystallogr.* **B59,** 393–403 (2003).
2. Jiang, H. *et al.* Molecular Crystal Engineering: Tuning Organic Semiconductor from p-type to n-type by Adjusting Their Substitutional Symmetry. *Adv. Mater.* **29,** 1605053 (2017).
3. Yoshida, H., Yamada, K., Tsutsumi, J. & Sato, N. Complete description of ionization energy and electron affinity in organic solids: Determining contributions from electronic polarization, energy band dispersion, and molecular orientation. *Phys. Rev. B* **92,** 075145 (2015).
4. Yamada, K. *et al.* Impact of the molecular quadrupole moment on ionization energy and electron affinity of organic thin films: Experimental determination of electrostatic potential and electronic polarization energies. *Phys. Rev. B* **97,** 245206 (2018).
5. Tietze, M. L. *et al.* Correlation of open-circuit voltage and energy levels in zinc-phthalocyanine: C60 bulk heterojunction solar cells with varied mixing ratio. *Phys. Rev. B* **88,** 085119 (2013).
6. Meiss, J. *et al.* Fluorinated Zinc Phthalocyanine as Donor for Efficient Vacuum-Deposited Organic Solar Cells. *Adv. Funct. Mater.* **22,** 405–414 (2012).
7. Schlettwein, D. *et al.* Electronic Energy Levels in Individual Molecules, Thin Films, and Organic Heterojunctions of Substituted Phthalocyanines. *J. Phys. Chem. B* **105,** 4791–4800 (2001).
8. Selzer, F. *et al.* Improved organic p-i-n type solar cells with n-doped fluorinated hexaazatrinaphthylene derivatives HATNA-F6 and HATNA-F12 as transparent electron transport material. *J. Appl. Phys.* **115,** 054515 (2014).
9. Chen, W. *et al.* Molecular Orientation Dependent Energy Level Alignment at Organic-Organic Heterojunction Interfaces. *J. Phys. Chem. C* **113,** 12832–12839 (2009).
10. Zhong, J. Q. *et al.* Effect of Gap States on the Orientation-Dependent Energy Level Alignment at the DIP/F16CuPc Donor-Acceptor Heterojunction Interfaces. *J. Phys. Chem. C* **115,** 23922–23928 (2011).
11. Duhm, S. *et al.* Orientation-dependent ionization energies and interface dipoles in ordered molecular assemblies. *Nat. Mater.* **7,** 326–332 (2008).
12. Graham, K. R. *et al.* The Roles of Structural Order and Intermolecular Interactions in Determining Ionization Energies and Charge-Transfer State Energies in Organic Semiconductors. *Adv. Energy Mater.* **6,** 1601211 (2016).
13. Chen, W., Qi, D., Gao, X. & Wee, A. T. S. Surface transfer doping of semiconductors. *Prog. Surf. Sci.* **84,** 279–321 (2009).
14. Bussolotti, F., Kera, S., Kudo, K., Kahn, A. & Ueno, N. Gap states in Pentacene Thin Film Induced by Inert Gas Exposure. *Phys. Rev. Lett.* **110,** 267602 (2013).
15. Han, W., Yoshida, H., Ueno, N. & Kera, S. Electron affinity of pentacene thin film studied by radiation-damage free inverse photoemission spectroscopy. *Appl. Phys. Lett.* **103,** 123303 (2013).

16. Schwarze, M. *et al.* Band structure engineering in organic semiconductors. *Science.* **352,** 1446–1449 (2016).

17. Schwarze, M. *et al.* Molecular Parameters Responsible for Thermally Activated Transport in Doped Organic Semiconductors. *Nat. Mater.* **18**, 242–248 (2019) .

18. Cnops, K. *et al.* Energy Level Tuning of Non-Fullerene Acceptors in Organic Solar Cells. *J. Am. Chem. Soc.* **137,** 8991–8997 (2015).
