# Supplementary material for: Impact of molecular quadrupole moments on the energy levels at organic heterojunctions
Source: Nat Commun. 2019 Jun 5;10:2466. doi: 10.1038/s41467-019-10435-2 (PMC6549189; doi:10.1038/s41467-019-10435-2)
Supplement: Supplementary file 2 — Peer Review File [file 41467_2019_10435_MOESM2_ESM.pdf]

Editorial Note: This manuscript has been previously reviewed at another journal that is not operating a transparent peer review scheme. This document only contains reviewer comments and rebuttal letters for versions considered at Nature Communications .

REVIEWERS' COMMENTS:

Reviewer #1 (Remarks to the Author):

The authors have appropriately addressed all of my concerns and the manuscript should be published in Nature Communications.

Reviewer #1 (Remarks to the Author):

Reviewer: *The authors have appropriately addressed all of my concerns and the manuscript should be published in Nature Communications.*

Author Reply: We thank the reviewer for the positive feedback and his/her support of the publication of our manuscript in Nature Communications.
